# Archaeal type IV pili and their involvement in biofilm formation

**DOI:** 10.3389/fmicb.2015.00190

**Published:** 2015-03-24

**Authors:** Mechthild Pohlschroder, Rianne N. Esquivel

**Affiliations:** Department of Biology, University of PennsylvaniaPhiladelphia, PA, USA

**Keywords:** biofilms, type IV pili, archaea, type IV pilins, adhesion

## Abstract

Type IV pili are ancient proteinaceous structures present on the cell surface of species in nearly all bacterial and archaeal phyla. These filaments, which are required for a diverse array of important cellular processes, are assembled employing a conserved set of core components. While type IV pilins, the structural subunits of pili, share little sequence homology, their signal peptides are structurally conserved allowing for *in silico* prediction. Recently, *in vivo* studies in model archaea representing the euryarchaeal and crenarchaeal kingdoms confirmed that several of these pilins are incorporated into type IV adhesion pili. In addition to facilitating surface adhesion, these *in vivo* studies also showed that several predicted pilins are required for additional functions that are critical to biofilm formation. Examples include the subunits of *Sulfolobus acidocaldarius* Ups pili, which are induced by exposure to UV light and promote cell aggregation and conjugation, and a subset of the *Haloferax volcanii* adhesion pilins, which play a critical role in microcolony formation while other pilins inhibit this process. The recent discovery of novel pilin functions such as the ability of haloarchaeal adhesion pilins to regulate swimming motility may point to novel regulatory pathways conserved across prokaryotic domains. In this review, we will discuss recent advances in our understanding of the functional roles played by archaeal type IV adhesion pili and their subunits, with particular emphasis on their involvement in biofilm formation.

## Introduction

Archaea and bacteria alike cope with stress by forming biofilms, multicellular communities encased in a structure consisting of polysaccharide layers (Monds and O’Toole, 2009; Haussler and Fuqua, 2013; Orell et al., 2013a). The initial steps in this process involve adherence to surfaces and interactions between cells (Wozniak and Parsek, 2014). A diverse set of surface filaments facilitate these interactions with biotic and abiotic surfaces. Within the bacterial domain, these filaments include the chaperone-usher pathway dependent pili, which are associated with the outer membranes of some gram-negative bacteria (Busch and Waksman, 2012) and the sortase-dependent cell wall-associated pili found in many gram-positive bacteria (Schneewind and Missiakas, 2013). Additionally, structurally conserved amyloid fibers consisting of autoaggregating polymeric fibrils that are composed of folded β-sheets have been identified in many bacterial phyla and may also play roles in archaeal biofilm formation (Blanco et al., 2012; Chimileski et al., 2014). The archaea also express a wide variety of additional, structurally diverse adhesion filaments. These filaments include the Mth60 fimbriae of *Methanothermobacter thermautotrophicus* (Thoma et al., 2008), 5 nm diameter filaments that adhere to organic surfaces such as chitin, the cannulae, 25 nm diameter hollow tubes that are found on the hyperthermophilic *Pyrodictium*, which mediate interactions between cells (Horn et al., 1999), and the hami of SM1 euryarchaea, which form grappling hooks that allow attachment to surfaces in cold sulfurous marshes (Perras et al., 2014).

Based on currently available genomic analyses and experimental data, only one known type of adhesion filament, the type IV pilus, appears to be present in nearly all phyla across both prokaryotic domains (Pohlschroder et al., 2011; Giltner et al., 2012; Szabo and Pohlschroder, 2012). These structures, which were first identified, and have been best studied in gram-negative bacteria, have been defined as filamentous protein complexes composed of subunits known as type IV pilins (Burrows, 2012a). The precursors of type IV pilins have N-terminal signal peptides that target them to the Sec pathway, which transports them across the plasma membrane where they are processed by a prepilin peptidase – either PilD, in bacteria, or PibD, in archaea – prior to incorporation into the pilus (Strom et al., 1993; Albers et al., 2003; Bardy and Jarrell, 2003). Unlike other Sec signal peptides, where the processing site follows the hydrophobic (H)-domain, the PilD/PibD processing site precedes the H-domain. The hydrophobic portions of these N-terminal domains, upon processing, serve as a scaffold at the core of the pilus to facilitate assembly. In addition to the conserved signal peptidases, the only components required for type IV pilus biosynthesis in all organisms appear to be PilB, an ATPase, and PilC, a transmembrane protein that has been proposed to anchor the pilus to the membrane (Nunn et al., 1990; Pelicic, 2008; Lassak et al., 2012; Takhar et al., 2013). Although additional components involved in pilus biosynthesis have been identified, none are conserved across the prokaryotic phyla.

In addition to surface adhesion, various type IV pili have evolved a variety of functions including nutrient scavenging (Zolghadr et al., 2011), mediating electron transport (Lovley, 2012), and facilitating protein transport across the periplasmic space of some gram-negative bacteria (Nivaskumar and Francetic, 2014). In fact, some type IV pilus-like structures have evolved in such a way that their functions no longer include surface adhesion. For example, the competence system of many gram-positive bacteria, which is critical for DNA uptake, has no effect on cell–cell interactions or surface adhesion, although assembly of these structures does require the type IV pilus biosynthesis machinery (Chen and Dubnau, 2004; Chen et al., 2006).

The development of several model archaeal systems (Leigh et al., 2011) as well as *in silico* tools that allow rapid, accurate identification of type IV pilins (Szabó et al., 2007), has allowed significant advances in the understanding of the roles played by type IV pili in the cellular processes of archaea. In this review, we will highlight recent advances that have been made in characterizing a diverse set of archaeal type IV pili, with particular attention paid to how these structures facilitate archaeal biofilm formation. These studies, which have focused primarily on a subset of type IV pili in the model crenarchaea, *Sulfolobus solfataricus* and *Sulfolobus acidocaldarius*, as well as the euryarchaeal model systems, *Methanococcus maripaludis* and *Haloferax volcanii*, have illuminated the strategies that have allowed organisms to thrive in the extreme environments that archaea often inhabit. The study of these evolutionarily conserved surface filaments in a diverse array of archaea has highlighted the divergent as well as the conserved aspects of type IV pili across a highly diverse range of prokaryotes, both within and across domains.

## Evolutionary Relationship between Type IV Pili and Type IV Pilus-Like Structures

### Type IV Pili

Biofilm formation is likely an evolutionarily ancient strategy adapted to protect microorganisms against highly stressful environmental conditions (Lopez et al., 2010). The presence of type IV pili in nearly all the phyla of bacteria and archaea (Szabó et al., 2007; Imam et al., 2011) indicates that these surface filaments are also ancient, perhaps being a prerequisite for the adaptations that allowed the establishment of these multicellular structures early on during the evolutionary history of the prokaryotes. The fact that most prokaryotic organisms still express type IV pili underscores the key roles they have played in the biological processes that allowed prokaryotes to flourish. Membrane-associated type IV pilins, pilus subunits, if not part of a pilus can promote adhesion in some organisms (Esquivel and Pohlschroder, 2014), suggesting that the earliest form of surface attachment was facilitated through membrane-anchored adhesion proteins that evolved to be incorporated into surface filaments, perhaps providing a mechanism that allowed cells to adhere more efficiently to surfaces.

Possibly reflecting the different types of surfaces to which type IV pili can adhere, the amino acid sequences of the pilins are highly diverse (Szabó et al., 2007; Imam et al., 2011). In fact, the only segment of the protein sequence that is largely conserved among all type IV pilins is limited to the unique prepilin-peptidase processing site in the N-terminal Sec signal peptide of pilin precursors (Giltner et al., 2012). The diversity of pilin sequences may also be due in part to the fact that many type IV pili have evolved to carry out additional functions. For example, *Geobacter sulfurreducens* expresses a type IV pilus that facilitates the transfer of electrons to extracellular electron acceptors such as insoluble metals (Reguera et al., 2005). Some additional pilus functions take advantage of the fact that many type IV pili have acquired the ability to retract, thus providing cells with a molecular ratchet that allows them to move along surfaces in a process that has come to be known as twitching motility (Bradley, 1972; Burrows, 2012b). The ability of pili to retract may also play a key role in maintaining close contact between cells while they exchange DNA (Ajon et al., 2011), and might also facilitate the uptake of viruses that attach to these surface structures (Kim et al., 2012). In some gram-negative bacteria, type IV pili, which are anchored to the inner membrane and cross the outer membrane through a secretin pore, can facilitate protein transport to the extracellular environment. For example, in *Vibrio cholera*, toxin co-regulated type IV pili are not only essential for surface adhesion, but are also required for secretion of the soluble colonization factor, TcpF (Kirn et al., 2003).

### Type IV Pilus-Like Structures

Some cell surface structures are assembled using homologs of the same components required for the biosynthesis of type IV pili, and share structural similarities with type IV pili, but have functions that do not include adhesion. For example, the piston-like structure of the type II secretion machinery of gram-negative bacteria, such as *Klebsiella oxytoca*, which is thought to facilitate the transport of proteins across the periplasm, is composed of type IV pilin-like proteins, and its substrates are secreted via pores composed of secretin (Giltner et al., 2012; Campos et al., 2013; Nivaskumar and Francetic, 2014). In fact, although this structure is not found on the cell surface of wild-type cells, the overexpression of its major piston subunit can result in the production of surface filaments (Sauvonnet et al., 2000; Nivaskumar and Francetic, 2014). In gram-positive bacteria, the assembly of a competence system that facilitates DNA uptake requires the presence of the PilD homolog, ComC, to process the subunits of a high molecular weight DNA-binding surface structure, as well as the PilB and PilC homologs, ComGA and ComGB, respectively. DNA binding pili have been visualized in *Streptococcus pneumoniae* (Laurenceau et al., 2013; Balaban et al., 2014). However, in the well-studied Com system of *Bacillus subtilis* these structures do not promote adhesion nor do they form surface filaments under the range of conditions that have been tested (Chen and Dubnau, 2004).

The key components required for the biosynthesis of archaeal flagella – rotating surface structures that drive swimming motility – are homologous to the core components of the type IV pilus-biosynthesis machinery (Jarrell and McBride, 2008). These structures are also composed of subunits that are processed by a prepilin peptidase, and the precursors of these flagellin subunits have signal peptides that are structurally similar to those of pilin precursors. It is likely that the flagella, which require several additional components to function properly (see review on flagella in this special issue), evolved from simpler type IV pili, a subset of which appears to promote surface adhesion in archaea, perhaps by overcoming surface tension barriers (Davey and O’Toole, 2000; Lassak et al., 2012). The lack of similarity between archaeal flagella and the flagella of bacteria has lead to a proposal to change the name of these structures to archaella (Jarrell and Albers, 2012). However, considering that flagella have not been defined based on their composition or the composition of their biosynthesis machineries, but rather by their function (Diniz et al., 2012), it seems fitting that these rotating surface structures remain known as flagella, just as these structures with analogous functions in bacteria and eukaryotes are. Consistent with this argument, the nomenclature used for pili seems to follow a similar logic. Pili are generally regarded as surface filaments that facilitate adhesion to biotic or abiotic surfaces. However, various types of pili, including the type IV pili, as well as others, such as the sortase-dependent pili, are named based on the composition of their biosynthesis machinery and structural subunits. Similarly, the name “archaeal flagella” clearly distinguishes these archaeal motility structures from the “bacterial flagella” which in turn are easily distinguished from the “eukaryotic flagella,” while these names still indicate that these are all surface structures that propel swimming motility.

Finally, a fascinating surface structure that is assembled using the same core pilus biosynthesis components described above is the *S. solfataricus* bindosome, which plays an important role in nutrient uptake (Zolghadr et al., 2007). The subunits of this structure, while containing processing sites that are typical of a type IV pilin, are at least four times the size of an average pilin and exhibit significant homology to substrate-binding proteins. Hence, these proteins are unlikely to have evolved from type IV pilins but rather were originally substrate-binding proteins that seem to have hijacked the type IV pilus biosynthesis machinery to assemble a surface structure that allows more efficient substrate scavenging.

*In silico* analyses have predicted a large number of genes encoding proteins that contain putative type IV pilin signal peptides in the genomes of species representing all archaeal phyla. These predicted proteins vary in size from less than 150 amino acids to well over 1000 (Szabó et al., 2007). Potential functions for most of these predicted proteins have not been identified, as this vast array of potential type IV pilus subunits has only recently begun to be characterized. As we learn more about the functions of these structures, we may also begin to tease out the evolutionary history of the type IV pili and their related structures.

## Pilus Biosynthesis

### Pilin Transport and Signal Peptide Processing

Type IV pilin precursors are transported across the cytoplasmic membrane via the Sec pathway in an SRP-dependent manner (Arts et al., 2007; Francetic et al., 2007). Like other Sec signal peptides, all type IV pilin signal peptides contain a charged N-terminus followed by a hydrophobic (H) domain. However, rather than following the H-domain, as in the signal peptides of other Sec substrates, the peptidase-processing site in the signal peptides of prepilins precedes it (Strom et al., 1993; Albers et al., 2003; Ng et al., 2009). Processing of the pilin precursors occurs either simultaneously with, or following, the lateral insertion of the H-domain into the cytoplasmic membrane, which, upon transport of the rest of the pilin through the Sec pore, may anchor the pilin to the membrane before it is incorporated into a pilus (Giltner et al., 2012).

In bacteria, the prepilin peptidase PilD is a bifunctional enzyme that cleaves and *N*-methylates type IV pilin precursors (Strom et al., 1993). While PilD exhibits only a low degree of homology with the archaeal prepilin peptidase, PibD, which lacks a region homologous to the bacterial protein domain required for pilin methylation, the prepilin peptidases of both domains are integral membrane aspartic acid proteases. Both proteases have similar catalytic sites, each containing two aspartic acids, of which the second one is part of a conserved GxHyD motif identified in many aspartic acid proteases (Szabo et al., 2006; Henche et al., 2014). Site-directed mutagenesis of the *Sulfolobales pibD* codons encoding the conserved aspartic acids resulted in mutant peptidases that are unable to process pilins as well as pilin-like subunits. Similarly, these conserved aspartic acids are required for processing of flagellins by the PibD homolog, FlaK, in *M. maripaludis* (Bardy and Jarrell, 2003; Szabó et al., 2007; Esquivel et al., 2013).

Although all type IV pilins and type IV pilin-like proteins share specific structural similarities, including a prepilin peptidase-processing site that precedes the H-domain, the importance of some commonly occurring characteristics within the tripartite structure of the signal peptide varies depending upon the particular prepilin peptidase involved in processing the pilin precursor (**Table 1**). For example, while proper processing of all type IV pilin precursors appears to require a G/A/S at the –1 position, a charged amino acid at position –2 is only critical in the recognition of archaeal pilin precursors by the prepilin peptidase (Szabó et al., 2007; Imam et al., 2011). Conversely, while the fifth amino acid of the mature bacterial pilin is nearly always a glutamate or aspartate, only a subset of archaeal pilins contain a negatively charged amino acid at the +5 position. Using these criteria, as well as a few additional conserved parameters, including the length and hydrophobicity of the H-domain of the signal peptide and the position of the processing motif, rule-based algorithms for genome-wide identification of genes that encode prepilins and prepilin-like proteins in bacteria (PilFind) and archaea (FlaFind) were successfully developed (Szabó et al., 2007; Imam et al., 2011; Esquivel et al., 2013).

**Table 1:** *In vivo* characterized archaeal type IV pilins.

Organism (# FlaFind pos.)	*Pilus*	*Pilin*	*PilB/PilC*	Prepilin peptidase		*N-glycosylation: Sequons/predicted ^4^*	*Assembly*	Role in biofilm formation		
				*Paralog*	*Processing site^3^*			*Adhesion*	*Microcolony formation*	*Positive regulation of swimming motility*
*M. maripaludis* (16)	**Epd**	EpdA	EpdL/EpdJ & K	EppA	R**A**QI	4/3	+/–	ND	ND	ND
		EpdB		EppA	K**G**QV	7/4	+	ND	ND	ND
		EpdC		EppA	K**G**QV	4/3	+	ND	ND	ND
		EpdD		EppA	K**G**QL	1/0	+	ND	ND	ND
		EpdE^1^		EppA	K**G**QI	4/3	+	ND	ND	ND
*S. acidocaldarius* (20)	**Ups**	UpsA^2^	UpsE/UpsF	PibD	K**A**IS	4/3	+	+/–	+^5^	ND
		UpsB^2^		PibD	K**G**IS	4/4	+	+/–	+^5^	ND
	**Aap**	AapA	AapE/AapF	PibD	R**A**LS	2/2	+	+	–	–
		AapB^1^		PibD	R**A**LS	4/3	+	+	–	–
*H. volcanii* (47)	**PilA**	PilA1^2^	PilB3/PilC3	PibD	D**A**VS	3/2	+	+	–	+
		PilA2^2^		PibD	D**S**AV	3/3	+	+	+/–	+
		PilA3^2^		PibD	D**A**VS	3/2	+	+	–	+
		PilA4^2^		PibD	R**A**VS	4/3	+	+	–	+
		PilA5^2^		PibD	R**A**VS	0/0	+	+	+	+
		PilA6^2^		PibD	R**A**VS	1/1	+	+	+	+

*^1^Determined as major pilins by mass spectrometry.*

*^2^Promote pilus biosynthesis in absence of other known pilins.*

*^3^Processing site is C-terminal to the bolded amino acid; predicted by FlaFind (Szabó et al., 2007).*

*^4^Number of Asn-Xaa-Ser/Thr sequons/# Predicted by Net-N-glyc 1.0.*

*^5^Aggregates formed in liquid media. See text for details.*

Most archaea express one prepilin peptidase that recognizes all prepilins as well as prepilin-like proteins such as the flagellins and bindosome subunits (Albers et al., 2003). However, a subset of euryarchaea expresses an additional, much larger, prepilin peptidase (EppA). Although substrates recognized by this second prepilin peptidase are predicted by FlaFind, they contain a highly conserved +1 glutamine, confirmed by top–down mass spectrometry (Ng et al., 2010), and the +5 glutamic acid as is commonly, and primarily, found in bacterial type IV pilins (Giltner et al., 2012). In fact, the *M. maripaludis* FlaK paralog, EppA, requires the presence of the +1 glutamine to process a pilin precursor, and it cannot process the *M. maripaludis* flagellin subunit (Szabó et al., 2007; Ng et al., 2009). Interestingly, while the PibD prepilin peptidases of *S. solfataricus* and *H. volcanii* appear to exhibit little stringency with regard to amino acid residues at positions +1 and +3 of the pilin precursors, the *M. maripaludis* FlaK does not process precursors having a +1 glutamine, preventing it from processing EppA substrates (Ng et al., 2009). It is likely that in other euryarchaea, which encode predicted EppA processed pilins, as well as PibD/FlaK processed pilins, the prepilin peptidases are similarly stringent. Expressing a single prepilin peptidase that processes both adhesion pilins and motility-promoting flagellins, as opposed to distinct prepilin peptidases for each, requires that distinct mechanisms regulate flagellin and pilin function. Details of some of these mechanisms have recently begun to emerge and are discussed below.

### *N*-Glycosylation

In bacteria, type IV pilins are often *O*-glycosylated and the components of the pathways involved in this protein modification have been well-characterized (Nothaft and Szymanski, 2010). This post-translational modification has been implicated in a variety of functions, including colony morphology, surface motility, and regulating pilus composition, with the specific roles played differing from species to species in bacteria (Smedley et al., 2005; Takahashi et al., 2012; Vik et al., 2012). *O*-glycosylation also affects how the immune systems of higher eukaryotes, such as humans, respond to invasive pathogenic species (Lizcano et al., 2012).

While *O*-glycosylation has not yet been reported for any archaeal pilins, some subsets of archaeal type IV pilins are predicted to be *N*-glycosylated (**Table 1**; Jarrell et al., 2014). A detailed description of the **a**rchaeal **gl**ycosylation (Agl) pathways is presented by Eichler in this special issue (Kandiba and Eichler, 2014). Briefly, sugars are initially assembled on a dolichol phosphate lipid carrier, are then “flipped” across the cytoplasmic membrane, and are finally transferred to the target protein by an oligosaccharyltransferase at a conserved Asn-X-Ser/Thr motif (Jarrell et al., 2014). Although the archaeal glycosylation pathways involved in glycosylation of pilin-like proteins contain a conserved AglB oligosaccharyltransferase (Abu-Qarn and Eichler, 2006; Chaban et al., 2006; VanDyke et al., 2009), the composition of the polysaccharide moiety added to the modified protein varies between species and even between the subunits of different surface filaments. For example, *M. maripaludis* flagellins are decorated with a tetrasaccharide that is similar to the pentasaccharide found on the type IV pilin EpdE, which contains an additional hexose attached as a branch to the linking GalNAc subunit (Ng et al., 2010). Interestingly, while *M. maripaludis* flagella processing by FlaK is not required for *N*-glycosylation it was recently shown that EppA-dependent signal peptide processing of EpdD and EpdE is required for the *N*-glycosylation of these pilins (Nair and Jarrell, 2015). Although glycosylation is required for the synthesis of the *M. maripaludis* flagella, its type IV pili appear to be stable in a Δ*aglB* strain (VanDyke et al., 2009). However, it remains to be determined whether the pili in this strain are functional. Thus far, with regards to glycosylation, only the deletion of the *M. maripaludis* acetyltransferase, which is required for the synthesis of the second sugar of the polysaccharide, affects proper cell surface attachment, as only few cell-associated pili could be identified in this strain, while culture supernatants contained pili (VanDyke et al., 2008).

The *H. volcanii* flagellins, FlgA1 and FlgA2, are also decorated with a pentasaccharide in an AglB-dependent manner; however, in this case, the pentasaccharide contains a hexose, mannose, two hexuronic acids, and a methyl ester of a hexuronic acid (Tripepi et al., 2012), the same that was previously identified on the *H. volcanii* s-layer (Abu-Qarn et al., 2007; **Table 1**). While the composition of polysaccharides attached to *H. volcanii* type IV pilins has not yet been determined, PilA1–PilA4 are also predicted to be *N*-glycosylated (**Table 1**). Interestingly, recent studies identified an alternative Agl pathway that differentially glycosylates the *H. volcanii* S-layer under low salt conditions (Guan et al., 2011). Furthermore, AglB-dependent glycosylation is diminished under these conditions (Kaminski et al., 2013). Hence, the *H. volcanii* pili might also display differential glycosylation under low salt conditions, although whether the glycosylation of these pili involves this newly identified pathway has yet to be elucidated. Preliminary data support *in silico* predictions suggesting that PilA1–PilA4 are *N*-glycosylated in an AglB-dependent manner and also show that these pilins probably inhibit microcolony formation that is promoted by PilA5 and PilA6 (Esquivel et al., 2013). Differential glycosylation of these adhesion pilins may be a regulatory mechanism that results in the inhibition of microcolony formation under stress conditions, such as low salt (see below). Since loss of AglB-dependent glycosylation of the flagellins also inhibits *H. volcanii* flagella biosynthesis (Tripepi et al., 2012), low salt conditions might promote biofilm formation by inhibiting flagella-dependent motility as well as alleviating the inhibition of microcolony formation that is dependent upon the glycosylation of PilA1–PilA4.

Thus far, only a small subset of pilins have been investigated for post-translational modifications; future molecular and cellular biological analyses combined with improving mass spectrometry methods, will undoubtedly reveal additional modifications and the roles they play in the biosynthesis, function, and regulation of archaeal type IV pili. Substantial progress has already been made in *Sulfolobales* in regards to flagellin glycosylation (Meyer and Albers, 2013), and it will be interesting to determine whether the crenarchaeal pilins are also glycosylated as predicted by *in silico* data (**Table 1**). A more thorough characterization of these modifications will lead to a better understanding of the mechanisms that regulate the biosynthesis and functions of these evolutionarily ancient surface filaments and may result in insights into the regulation of biofilm formation and dispersal under extreme conditions.

### Major and Minor Pilins

**FIGURE 1:**
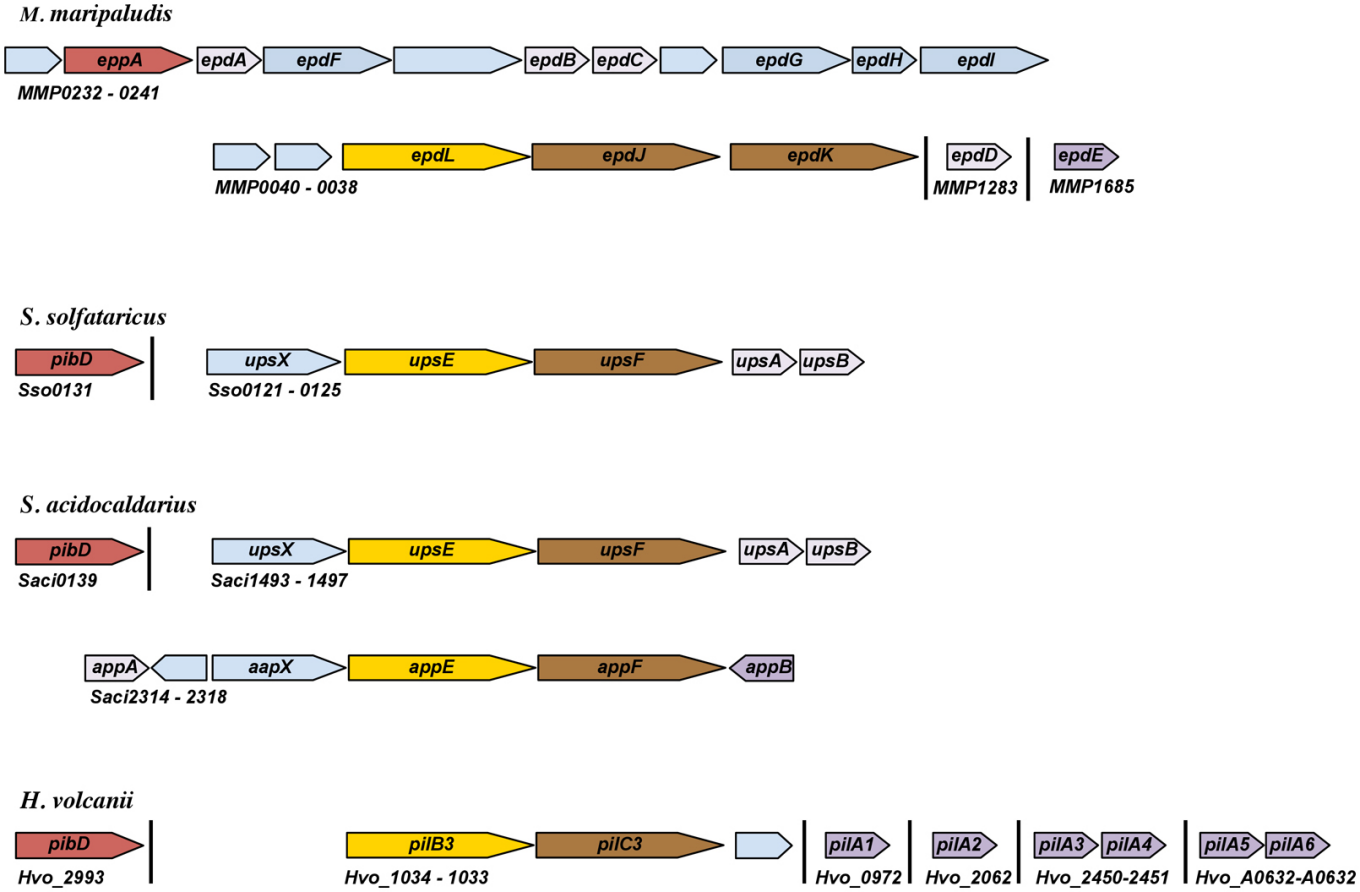
Schematic representation of genomic loci encoding pilins and pilus biosynthesis components. Arrows represent relative orientation of open reading frames (ORFs); ORFs of the same color correspond to genes with similar function. Annotation is based on experimental characterization as described in the text or as recorded by NCBI. Hypothetical proteins are light blue. Major pilins, as determined by mass spectrometry or whether the pilin can promote pilus biosynthesis in the absence of other known pilins, are dark purple. Minor pilins that have been experimentally characterized are light purple.

In any given archaeon the number of genes that encode predicted PibD-processed substrates can vary greatly (**Table 1**). While many of these genes likely encode the subunits of specific pilus-like structures, depending on growth conditions, an archaeon might produce differing sets of type IV pili. As in bacteria (Kuchma et al., 2012), archaeal pili can be composed of major and minor pilins. For example, while EpdE, as determined by Mass spectrometry, is the major pilin of *M. maripaludis*, two additional pilins, EpdB, and EpdC, are also required for piliation, and cells lacking EpdA have reduced piliation under the conditions tested. The exact functions of these minor pilins, whose genes are co-regulated with pilus biosynthesis genes, are largely unknown (**Figure 1**; Ng et al., 2010). Six additional genes encoding putative minor pilins *mmp0528, mmp0600, mmp0601, mmp0709, mmp0903,* and *mmp1283 (epdD)* were recently investigated to determine whether the proteins they encode are involved in pilus assembly Investigations of specific deletion mutants for each gene determined that only the deletion of *epdD* results in the loss of piliation (Nair et al., 2014). While pili containing EpdE as the major pilin appear to be the only type IV pili that are synthesized in this methanogen under the standard laboratory conditions tested, it is unclear whether the other predicted pilins might be involved in related functions, such as regulating pili-assembly or perhaps in forming distinct pili under different conditions or when attached to different surfaces rather then expressed in planktonic cells (see below).

The differential expression of pilins has indeed been demonstrated in *S. solfataricus,* which encodes 28 FlaFind positives (Szabó et al., 2007). However, the only known type IV pilus produced by *S. solfataricus* is induced through exposure to ultraviolet (UV) light. This UV-inducible pilus, known as Ups, which plays important roles in both aggregation and surface attachment, is believed to be composed of UpsA and UpsB, two pilins encoded by genes adjacent to the pilus-biosynthesis genes *upsE* and *upsF* (**Figure 1** and see below). When overexpressing these pilins, *S. solfataricus* produces long, irregular pili (Fröls et al., 2008). Consistent with each of them being major pilins, individual deletions of these homologous genes in *S. acidocaldarius* still results in piliation, although fewer pili are observed (van Wolferen et al., 2013). Whether these pilins form mixed pili in wild-type cells is not known. Perhaps additional pilins, encoded by genes that are not co-regulated with the biosynthesis genes, are required for pilus formation, as demonstrated in *M. maripaludis*. Of the additional 24 FlaFind positive genes in *S. solfataricus*, one encodes the flagellin and three encode known substrate-binding proteins, the functions of the remaining 20 predicted PibD substrates remain elusive.

In addition to the Ups pili, *S. acidocaldarius* also produces the archaeal adhesive or Aap pilus, which plays a major role in surface adhesion (Henche et al., 2012a). The function and assembly of the Aap pilus requires the presence of at least two pilins, AapA and AapB, both of which are encoded by genes located adjacent to Aap pilus biosynthesis genes, *aapE* and *aapF* (**Figure 1** and see below). Unlike the Ups pili, the deletion of either gene encoding these Aap pilin subunits results in the absence of Aap pili. As only AapB was identified by mass spectrometry of purified Aap filaments, AapB appears to be the major pilin under the tested conditions (Henche et al., 2012a). Interestingly, while *aapB* expression is downregulated during stationary phase, *aapA* expression is increased, suggesting that the composition of the pili produced by cells varies depending upon growth conditions. As in *S. solfataricus*, aside from the pilins *aapA* and *aapB*, the eight substrate-binding proteins and one flagellin, the functional roles of most of its 20 FlaFind positives are not known.

**FIGURE 2:**
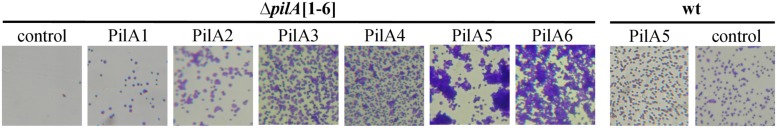
A subset of *H. volcanii* adhesion pilins promotes microcolony formation while a distinct set appears to inhibit this process. Liquid cultures of *H. volcanii* Δ*pilA[1-6]* or wild-type with an expression vector (control) or the plasmid expressing individual His-tagged PilA pilins, were incubated overnight in liquid media with plastic cover slips to assay surface adhesion using the modified Ali assay (O’Toole and Kolter, 1998; Tripepi et al., 2010). Light micrographs of the coverslips taken at 35× magnifications are shown. Modified image from Esquivel et al. (2013).

Interestingly, in *H. volcanii*, the deletion of six adhesion pilin genes *pilA1-pilA6*, none of which is co-regulated with pilus-biosynthesis genes, is required to inhibit pilus-formation (**Table 1** and **Figures 1 and 2**; Esquivel et al., 2013). Among the 47 predicted PibD substrates of *H. volcanii*, only these six pilins contain an almost completely conserved H-domain, PilA2 containing one additional N-terminal serine. Each of these six pilins can form a functional type IV pilus when expressed individually in a Δ*pilA*[*1-6*] strain, suggesting that each can serve as the major pilin. However, it should be noted that, while pili in a wild-type *H. volcanii* strain are 8–12 nm in width and can be up to 4 μm long, individually expressed PilA1–PilA6 in Δ*pilA*[*1-6*] make very short pili (Esquivel et al., 2013). Preliminary data strongly indicate that only a subset of these six pilins is expressed during planktonic growth while another set is expressed under sessile conditions. The critical importance of the pilin H-domain for pilus-biosynthesis was demonstrated by the fact that a fusion protein PilA1Hybrid, in which the conserved pilin H-domain is replaced with an unrelated H-domain, cannot form pili in the Δ*pilA*[*1-6*] strain (Esquivel et al., 2013; see below).

### Pilus-Assembly

Throughout prokaryotes, assembly of type IV pili occurs using energy obtained by the evolutionarily conserved ATPase, PilB. While it is known that the hydrolysis of ATP by this VirB11 ATPase provides the energy, it is not known how this energy is transferred to the pilin and how the pilin is moved from the membrane into the pilus. While the transmembrane protein, PilC, is proposed to be the membrane anchor for the pilus, to date there is no definitive evidence for this function. However, genes encoding either of these evolutionarily conserved biosynthesis components are essential for piliation (**Table 1**; Pelicic, 2008).

*M. maripaludis* type IV pili, which have a diameter of ∼6 nm, have a hollow lumen unlike other type IV pili with available structures. Similar to bacterial pili, they require a *pilB* homolog (*epdL*) for assembly. Moreover, *pilB* is co-regulated with two *pilC* paralogs (*epdJ* and *epdK*), which are both essential for piliation (Hendrickson et al., 2004; Nair et al., 2014). The requirement for two PilC paralogs is reminiscent of the requirement for two co-regulated *pilC* paralogs, *tadB* and *tadC* in *Aggregatibacter actinomycetemcomitans* (Kachlany et al., 2000). It has been proposed that the single ATPase of the Tad system may interact with one version of the conserved membrane proteins in pilin addition and with the other in pilin removal (Burrows, 2012b). However, whether archaeal type IV pili can retract has not yet been determined (see below). It should be noted that, while an archaeal retraction ATPase, PilT, has not been identified, *M. maripaludis*, in addition to EpdL and FlaI, does encode one additional VirB11-like ATPase, which, like PilT, is not encoded by a gene co-localized with a *pilC* (Nair et al., 2014). While this protein is not required for pilus-biosynthesis in *M. maripaludis*, it remains to be determined whether it is involved in retraction.

Unlike the *M. maripaludis* genome, which appears to contain only a single *fla* and a single *pil* biosynthesis operon, many archaea, despite encoding only one, or, at most, two PibD paralogs, contain several operons that encode PilB and PilC homologs. The *S. solfataricus* genome*,* for example, contains the *bas* operon, which encodes among other proteins, the PilB and PilC homologs that are required for the incorporation of substrate-binding proteins into a bindosome, as well as the previously noted *ups* operon, which encodes UpsE and UpsF, PilB and PilC homologs, respectively (She et al., 2001; Zolghadr et al., 2007; van Wolferen et al., 2013). While wild-type cells, upon UV-induction form pili of up to 16 μm in length that have a diameter of approximately 10 nm, the deletion of *upsE* results in a non-piliated strain (Fröls et al., 2008). At least a subset of the *ups* pilins are also encoded by this operon, as noted above. *S. acidocaldarius* shares the *fla*, *bas* and *ups* operons with *S. solfataricus*, but also has an additional *pil* operon that encodes the PilB and PilC paralogs, AapE and AapF, respectively (Chen et al., 2005). Cells lacking either AapE or AapF do not form Aap pili, long filaments, 8–10 nm in diameter, that unlike the *M. maripaludis* pili, are not hollow (Henche et al., 2012a). Interestingly, a strain expressing AapE, but lacking UpsE, fails to assemble Ups pili upon UV-induction, suggesting that neither PilB paralog can complement the function of the other (Henche et al., 2012b). Similarly, in *H. volcanii*, which contains five putative *pil* operons, a *pilB3-C3* deletion strain, which lacks the PilB and PilC homologs required for assembling PilA1–PilA6 pili, does not have pili on the cell surface (Esquivel and Pohlschroder, 2014). While microarray data have indicated that three of the five *pilBpilC-*containing operons are not transcribed at detectable levels under experimental conditions tested to date, *pilB4* and *pilC4* are expressed under these conditions. The lack of PilA pilus formation in the Δ*pilB3-C3* background suggests that PilB4 and PilC4 are specifically involved in the biosynthesis of a pilus-like structure composed of two relatively large pilins encoded by FlaFind positive genes that are co-regulated with these pilus-biosynthesis components (Szabó et al., 2007).

Similar to *M. maripaludis*, the genomes of both *Sulfolobales* strains examined, as well as *H. volcanii*, encode a PilB homolog that is not co-regulated with a PilC homolog (Hartman et al., 2010). Perhaps these “orphan” PilB homologs are involved in pilus retraction, similar to PilT in bacteria. Although, as noted above, twitching motility has not yet been observed in any archaea, a recent report has shown evidence for *H. volcanii* social motility, where waves of this haloarchaeon were observed moving through a liquid medium (Chimileski et al., 2014). While this differs from surface motility, type IV pili may be required for this process, as is true for the bacteria *Stigmatella aurantiaca* and *Myxococcus xanthus* (Tan et al., 2013).

Similarly, following initial *H. volcanii* attachment where an even distribution of cells is seen across a surface, the flagella-independent transition into microcolonies may also require type IV pili retraction (Esquivel et al., 2013). Determination of the effect that deleting the *H. volcanii* “orphan” *pilB* homolog has on these functions may lead to intriguing new lines of inquiry into the roles played by type IV pili in a variety of archaeal cellular processes.

A number of well-studied gram-negative pilus-biosynthesis components, such as PilQ, an outer membrane pore known as a secretin, as well as additional components required for the biosynthesis of this pore, are not found in monoderm archaea (Ayers et al., 2010). Moreover, as noted, several types of post-translational modifications of type IV pilins, such as methylation and *O*-glycosylation, have been identified in bacteria (Nothaft and Szymanski, 2010; Kim et al., 2011). Unlike PilD, as noted above, the archaeal prepilin peptidase, PibD, is not a methyltransferase (Albers et al., 2003) and neither methylated pilins, nor a methyltransferase that specifically methylates archaeal pilins, have thus far been identified. However, the analysis of archaeal type IV pilin post-translational modifications has been limited and *in vivo* studies, rather than *in silico* analyses, are more likely to identify any novel archaeal biosynthesis components. Two examples are the identification of the AglB-dependent type IV pilin *N*-glycosylation, which appears to be limited to the archaea (Jarrell et al., 2014) as well as AapX, a protein required for biosynthesis of the *S. acidocaldarius* Aap pili, but whose exact role is still elusive (Henche et al., 2012a).

## Roles of Type IV Pili and its Pilins in Biofilm Formation

Type IV pili play important roles in several processes required for surface-associated biofilm formation, including surface attachment and microcolony formation, as well as the aggregation of cells in liquid media (Giltner et al., 2012; Lassak et al., 2012; Esquivel and Pohlschroder, 2014). Minor pilins also play crucial roles in regulating the assembly of pili in bacteria (Cisneros et al., 2012; Nguyen et al., 2015). Archaea have been identified in biofilms established in a diverse variety of microbial ecosystems, from acidic hot spring mats to methane-rich marine sediments and hypersaline lakes (Whitaker et al., 2005; Coman et al., 2013; Aoki et al., 2014). Several model systems of archaea, including *M. maripaludis* and *H. volcanii,* as well as the *Sulfolobales* species, *S. acidocaldarius* and *S. solfataricus*, can also form biofilms (Fröls et al., 2012; Henche et al., 2012b; Fröls, 2013; Brileya et al., 2014). Recent molecular biological analyses of biofilm formation in these organisms have revealed that archaeal type IV pili, like their evolutionarily conserved bacterial counterparts, are involved in surface adhesion and cell aggregation (Jarrell et al., 2011; Henche et al., 2012a; Esquivel et al., 2013). Moreover, archaeal pilins also seem to be involved in regulating microcolony formation and flagella-dependent motility, functions that, while previously unrecognized, might be broadly conserved across the prokaryotes (Esquivel and Pohlschroder, 2014).

### Adhesion to Abiotic Surfaces

*M. maripaludis* can attach to a diverse set of materials, including glass, nickel, gold, silicon, and molybdenum, as has been determined by electron microscopy (**Table 1**; Jarrell et al., 2011). Attachment of cells in shaking cultures to all the abiotic surfaces tested thus far is EppA-dependent. However, it has not yet been determined which of the nine predicted methanogen pilins, or combination thereof, are required for attachment to these different surfaces. While cells grown in liquid media appear to have pili composed of the major pilin EpdE, under the conditions where cells adhere to various surfaces the composition of the pili may be distinct (Jarrell et al., 2011). While 14 *M. maripaludis* FlaFind positive pilins are predicted to be processed by EppA, only five of these have thus far been shown to be required for pilus assembly under the conditions tested (Ng et al., 2010; Nair et al., 2014).

**FIGURE 3:**
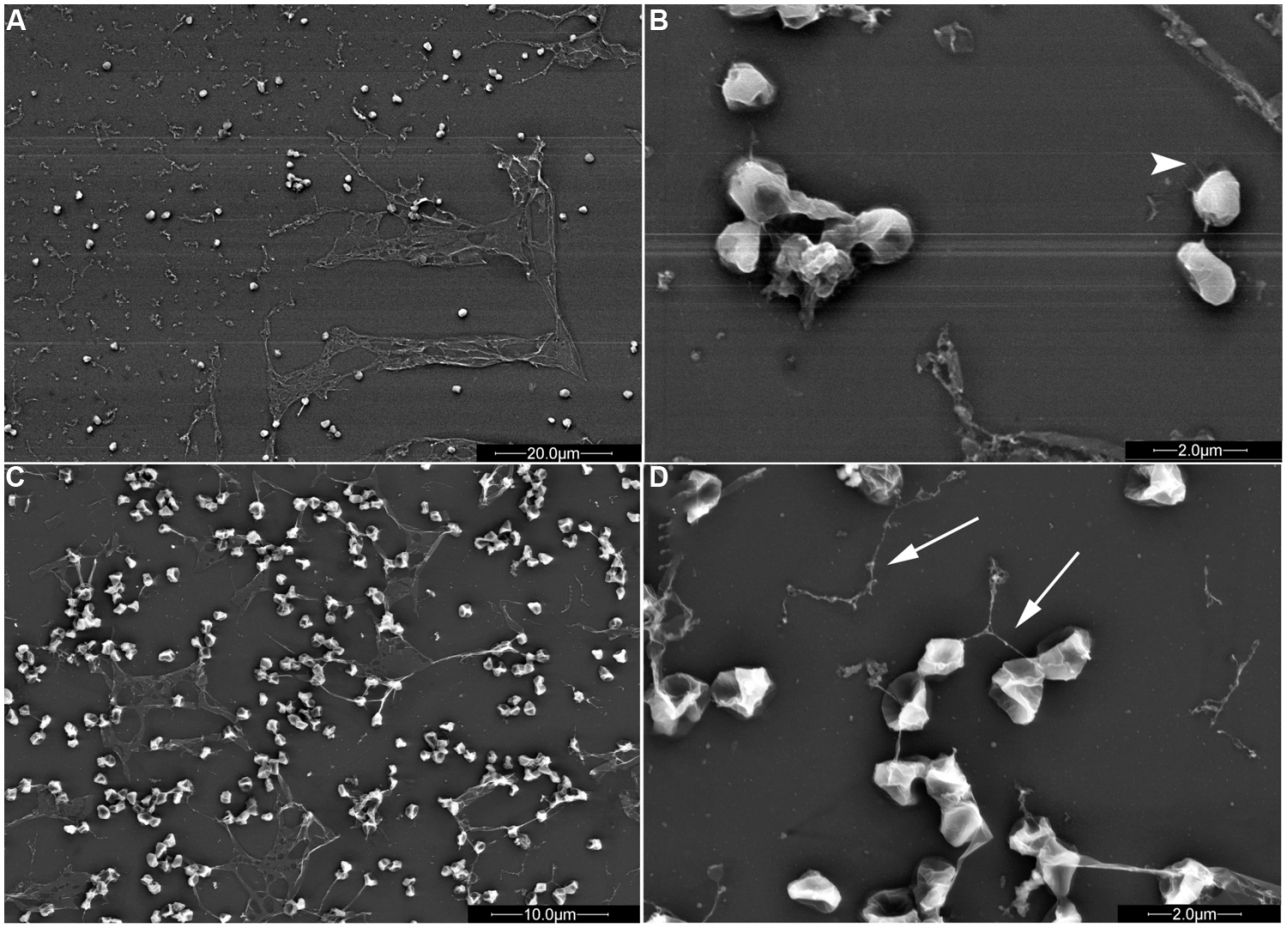
*S. solfataricus* adhesion patterns differ depending on the surface. *S. solfataricus* cells attached to mica (**A,B**; **B** is an enlargement of **A**) produce significantly more sheath-like structures compared to cells attached to glass (**C,D**; **D** is an enlargement of **C**). Pili (arrowheads) and flagella (arrows) are indicated. Bars: 20 μm **(A)**, 2 μm **(B,D)**, and 10 μm (**C**; Zolghadr et al., 2009). Mica or glass surfaces were incubated in shaking cultures for 2 days in liquid medium, followed by electron microscopy to observe surface adhesion. Image courtesy of Sonja Albers, University of Freiburg.

Type IV pilus-dependent adhesion to different surfaces has also been demonstrated for the *Sulfolobales* model organisms using shaking cultures. While wild-type *S. solfataricus* can attach to mica, glass, pyrite, and carbon-coated gold grids (**Figure 3**), the *S. solfataricus upsE* deletion mutant will not adhere to any of these surfaces. Type IV pili are more highly expressed during growth on a surface as determined by electron microscopy of cells attached to the four surfaces (Zolghadr et al., 2009). Whether the known Ups pili subunits, UpsA and UpsB are crucial for adhesion to the various surfaces noted above is not yet known. Interestingly, the patterns of *S. solfataricus* attachment to a surface are distinct, depending upon the specific material, perhaps indicating that the pili in each case have a distinct pilin composition.

Consistent with *S. acidocaldarius* containing a second adhesion pilus, deleting *upsE* does not lead to defective adhesion to glass in this closely related crenarchaeon, nor does deleting the gene encoding the *S. acidocaldarius* membrane protein AapF affect adhesion to this surface (Henche et al., 2012b). However, consistent with at least one of these pili being required for adhesion, a Δ*upsE*Δ*aapF* strain has a severe adhesion defect. Interestingly, a Δ*aapF*Δ*flaJ* strain also exhibits a defective adhesion phenotype, supporting previous studies that indicated a role for *Sulfolobales* flagella in adhesion (Zolghadr et al., 2009; Henche et al., 2012b). While flagella also appear to play a role in surface adhesion in *M. maripaludis* (Jarrell et al., 2011), the *H. volcanii* flagella do not. Adhesion was assessed by the accumulation of cells at the air-liquid interface of a glass coverslip incubated in static liquid culture (O’Toole and Kolter, 1998; Tripepi et al., 2010). Perhaps a flagella-driven force is not necessary to overcome the lower surface tensions that are found under high salt conditions compared to the surface tension that most prokaryotes must master in order to maintain contact with a surface during attachment (O’Toole et al., 2000). However, *H. volcanii* does require PilA1–PilA6 for attachment to a glass or plastic surface (Esquivel et al., 2013). Surprisingly, cells of the non-adhering Δ*pilA*[*1-6*] strain not only produce pili when expressing any of the individual pilins in *trans*, their ability to adhere to glass and plastic is also restored, consistent with the ability of each of these pilins to form a functional pilus. Interestingly, the ability of these cells to adhere to both surfaces varies depending on the pilin expressed. For instance, cells expressing either PilA1 or PilA2 have the lowest affinity for a glass or plastic surface under the conditions tested (**Figure 2**). PilA1 and PilA2 might be better adapted for binding to surfaces that are more frequently encountered in the natural environment by *H. volcanii.* Halophilic archaea have been isolated from brine shrimp, suggesting that chitin could be an ideal surface on which to test the adhesive capabilities of the six *H. volcanii* adhesion pilins (Riddle et al., 2013). Similar differential adhesive properties have been noted for specific pili in other species, including *V. cholerae*, which uses the MshA pilus to adhere to chitin and the TcpA pilus to attach to epithelial cells (Watnick et al., 1999; Krebs and Taylor, 2011).

Finally, Δ*pilA*[*1-6*] cells expressing a PilA1Hybrid in *trans*, in which the conserved H-domain of the pilin is replaced with an unrelated hydrophobic domain, are unable to adhere to glass, consistent with the inability of these cells to assemble pili (see above; Esquivel et al., 2013). Additionally, the Δ*pilB3-C3* strain, which lacks the core biosynthesis components required for PilA pilus biosynthesis, retains some ability to adhere to glass, indicating that membrane-associated pilins can promote some surface adhesion (see above). Thus, the lack of adhesion by Δ*pilA*[*1-6*] cells expressing the PilA1Hybrid indicates that the H-domain has an additional role other than proper pilus assembly.

It will be intriguing to discover additional surfaces to which these varied archaea can adhere, and to determine whether the expression of as yet uncharacterized type IV pili or perhaps a distinct combination of pili or pilins or both is required to complete this crucial initial step in the formation of biofilms on these surfaces. Different environmental conditions may also induce the expression of a distinct set of pili. Although archaeal biofilms have been observed in natural environments and examined for composition (Wilmes et al., 2008), little work has been done to identify the archaeal type IV pili that are expressed in the cells that inhabit these natural environments or what additional functions these structures might perform in multispecies communities.

### Cell Aggregation and Microcolony Formation

Biofilm formation is typically initiated by cells adhering to an abiotic surface followed by the formation of type IV pilus-dependent cell aggregates called microcolonies, which are encased in a polysaccharide matrix (Monds and O’Toole, 2009; Haussler and Fuqua, 2013; Orell et al., 2013a). While wild-type microcolony formation is not apparent even after 24 h, an *H. volcanii* Δ*pilA*[*1-6*] strain expressing either PilA5 or PilA6 in *trans* will form microcolonies after about 8 h (Esquivel et al., 2013). Since heterologous expression of either of these pilins in wild-type cells does not promote microcolony formation, at least a subset of the remaining adhesion pilins, PilA1–PilA4, must inhibit the formation of these cell aggregates. Not only is this the first indication that a specific subset of pilins can promote microcolony formation, it is also the first time that a distinct subset of pilins has been suggested to inhibit it. This regulatory mechanism might prevent cell aggregation of planktonic cells that express a subset of pili, allowing cells to quickly attach to surfaces when necessary. Whether the cell aggregation that results in microcolony formation requires retractable type IV pili or is accomplished through another mechanism is not yet known.

Pilus-dependent cell-to-cell interactions have been demonstrated for *S. solfataricus* and *S. acidocaldarius* in liquid media, where the Ups pili promote the formation of cell aggregates upon UV exposure (Fröls et al., 2008; Ajon et al., 2011). In this case, cellular aggregation, which is required for DNA exchange between the cells of these two species, is believed to be part of a response to DNA damage. In addition, these aggregates might be precursors of floating, pellicle biofilms that can protect cells from UV light as well as other stresses. While, as described above, unlike Δ*upsE* strains, which lack type IV pili, Δ*upsA* and Δ*upsB* strains, when analyzed by EM still had some pili; however, the UV-induced aggregation defect was similar in all strains, suggesting that the remaining pili could not significantly promote this cell–cell interaction (van Wolferen et al., 2013). It is not yet known whether the composition of Ups pili required for UV-induced aggregation in liquid media and those involved in adhesion to abiotic surfaces (see above) are identical.

*H. volcanii* can also exchange DNA through mating (Rosenshine et al., 1989) and biofilm formation promotes this DNA exchange (Chimileski et al., 2014). Interestingly, under non-biofilm conditions this process is not dependent on type IV pili, as deleting *pibD* does not affect mating despite abolishing pilus-assembly and surface adhesion (Tripepi et al., 2010).

### Biofilm Maturation

The maturation of a surface biofilm, following surface adhesion and microcolony formation, results in large cell aggregates encased in exopolysaccharide (EPS) with differing morphologies between species (Fröls, 2013). The effects of type IV pili on the maturation of an archaeal biofilm have only recently been examined and are limited to studies in the *Sulfolobales*.

**FIGURE 4:**
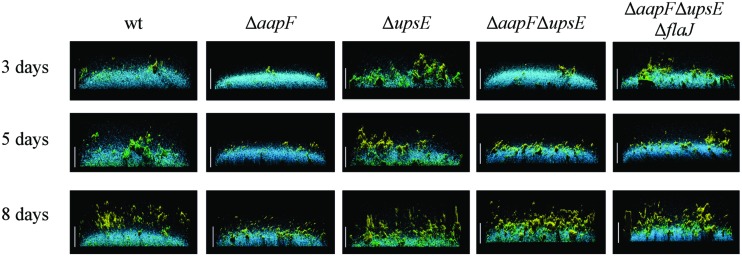
The loss of distinct type IV pili results in different biofilm structures. Side view of *S. acidocaldarius* biofilms after 3, 5, and 8 days of incubation. The blue channel represents DAPI staining. The green channel shows fluorescent labeled lectin ConA, which binds to glucose and mannose residues. The lectin IB4 binds galactosyl residues and is shown in yellow. Scale bar = 40 μm (Henche et al., 2012b). Image courtesy of Sonja Albers, University of Freiburg.

*S. solfataricus* liquid cultures grown in petri dishes form low-density carpets, covering the entire surface of the petri dish after 3 days of incubation. Thin cell-to-cell connections between aggregates within these biofilms have been observed by confocal liquid scanning microscopy. These connections can be stained by GSII, which binds *N*-acetylglucosamine residues, suggesting that these thin connections may be formed by glycosylated type IV pili (Koerdt et al., 2010).* S. acidocaldarius* wild-type cells, under similar growth conditions, establish thicker biofilms in which towering cell aggregates form, leaving some uncovered space between aggregates. An *S. solfataricus upsE* mutant strain, forms a significantly less dense biofilm, with an increase in the number of cell aggregates (Koerdt et al., 2010). Similar to *S. solfataricus,* a *upsE* deletion mutant in *S. acidocaldarius* forms a less dense biofilm that consists of loose aggregates, although these biofilms still maintain high, tower-like structures (**Figure 4**; Henche et al., 2012b). Staining of the biofilms with ConA and IB4 lectins, which bind to mannose/glucose and galactosyl sugar residues, respectively, has suggested that EPS production of these sugars is highly induced in the *upsE* deletion strain of *S. acidocaldarius*. Contrary to the phenotype seen upon loss of the Ups pilus, the deletion of *aapF* in *S. acidocaldarius* results in a denser biofilm with a decreased thickness and lacking the towering structures (Henche et al., 2012a,b). These results suggest that the Aap pili might be involved in maintaining a certain distance between the cell aggregates in the biofilm, perhaps to allow for optimal nutrient flow. Conversely, it is conceivable that the Aap pili facilitate surface (twitching) motility, which promotes microcolony formation following initially uniform adhesion to the surface. Hence, in the absence of these pili, cells are unable to form microcolonies and will rather expand evenly into a dense biofilm. Alternatively, the deletion of Ups pili may inhibit microcolony formation.

A subset of *H. volcanii* type IV pili promotes microcolony formation while another distinct set of pilins inhibits this type of cell aggregation, which is consistent with the observation that, while *H. volcanii* form microcolonies 2 days after inoculation into static liquid cultures (Chimileski et al., 2014), a Δ*pilA*[*1-6*] strain expressing either PilA5 or PilA6, begins forming microcolonies within 8 h of inoculation (Esquivel et al., 2013). As discussed above, it may be that biofilms mature more quickly when PilA1–PilA4 are not expressed. Cells expressing only PilA1 or PilA2 do not adhere as well-compared to the wild-type while cells expressing either PilA3 or PilA4 appear to adhere better, but they do not form microcolonies. *H. volcanii* biofilms observed after at least 7 days of incubation, form tall cell aggregate towers having a flaky appearance (Fröls et al., 2012; Chimileski et al., 2014). It will be intriguing to determine whether, similar to the *S. acidocaldarius aap* mutants, the mature *pilA*[*5-6*] mutant *H. volcanii* biofilm is denser. Much like the *Sulfolobales* biofilms, staining with ConA reveals EPS in this haloarchaeal biofilm. Interestingly, congo red also stains 3 day old biofilms, indicating the presence of amyloid protein. Since cells in mature biofilms behave differently than during biofilm formation, it is important to continue to examine the roles type IV pili play in maintaining, as well as forming, a biofilm.

### Regulation of Flagella-Dependent Motility

To initiate biofilm formation, both bacteria and archaea must have regulatory mechanisms that facilitate the transition of cells from a planktonic to a sessile state when local conditions warrant it (McDougald et al., 2011). In the planktonic state, prokaryotic cells produce functional flagella that allow them to move through the environment, seeking nutrients and avoiding unfavorable conditions, while sessile cells use type IV pili to attach to abiotic surfaces and form microcolonies (O’Toole and Kolter, 1998; Ghosh and Albers, 2011). Thus, there exists an inverse relationship in these states between the expression of functional flagella and type IV pili and the respective sets of genes that encode components of the biosynthesis machinery for each (Fröls et al., 2008; Karatan and Watnick, 2009; Pohlschroder et al., 2011; Esquivel et al., 2013). Deleting either the gene encoding the major pilin *aapB* or pilin biosynthesis genes *aapE* and *aapF* results in hypermotility in the crenarchaeon *S. acidocaldarius* (Henche et al., 2012a). Quantitative RT-PCR has revealed that deleting *aapF* leads to an increase in the expression of the flagellin gene, *flaB,* and the flagella gene *flaJ*, encoding a PilC homolog. This result is consistent with the hypermotility phenotype observed for the *aapF* deletion mutant (Henche et al., 2012a,b). Deleting *upsE* also leads to an increase in the expression levels of the *fla* genes, albeit somewhat less significantly (Henche et al., 2012b). The expression levels of these genes seem to be linked through a regulatory mechanism, which is not surprising given their roles at different stages during the formation, maintenance and dispersal of biofilms. However, in bacteria, where a similar inverse expression has also been observed, regulation of the changes in expression of the proteins involved in the assembly and function of the pili and flagella is often controlled by local concentrations of cyclic-di-GMP (Bordeleau et al., 2014; Martinez and Vadyvaloo, 2014). While c-di-GMP has not been shown to play a role in regulating archaeal pili and flagella expression, a recent study in *S. acidocaldarius* demonstrated that the deletion of a gene encoding the Lrs14 transcription regulator, Saci0446, significantly upregulates *aapA* expression and increases biofilm formation while at the same time downregulating *flaB* and causing impaired motility (Orell et al., 2013b). Thus far, additional specific molecules that regulate biofilm formation in archaea are unknown.

**FIGURE 5:**
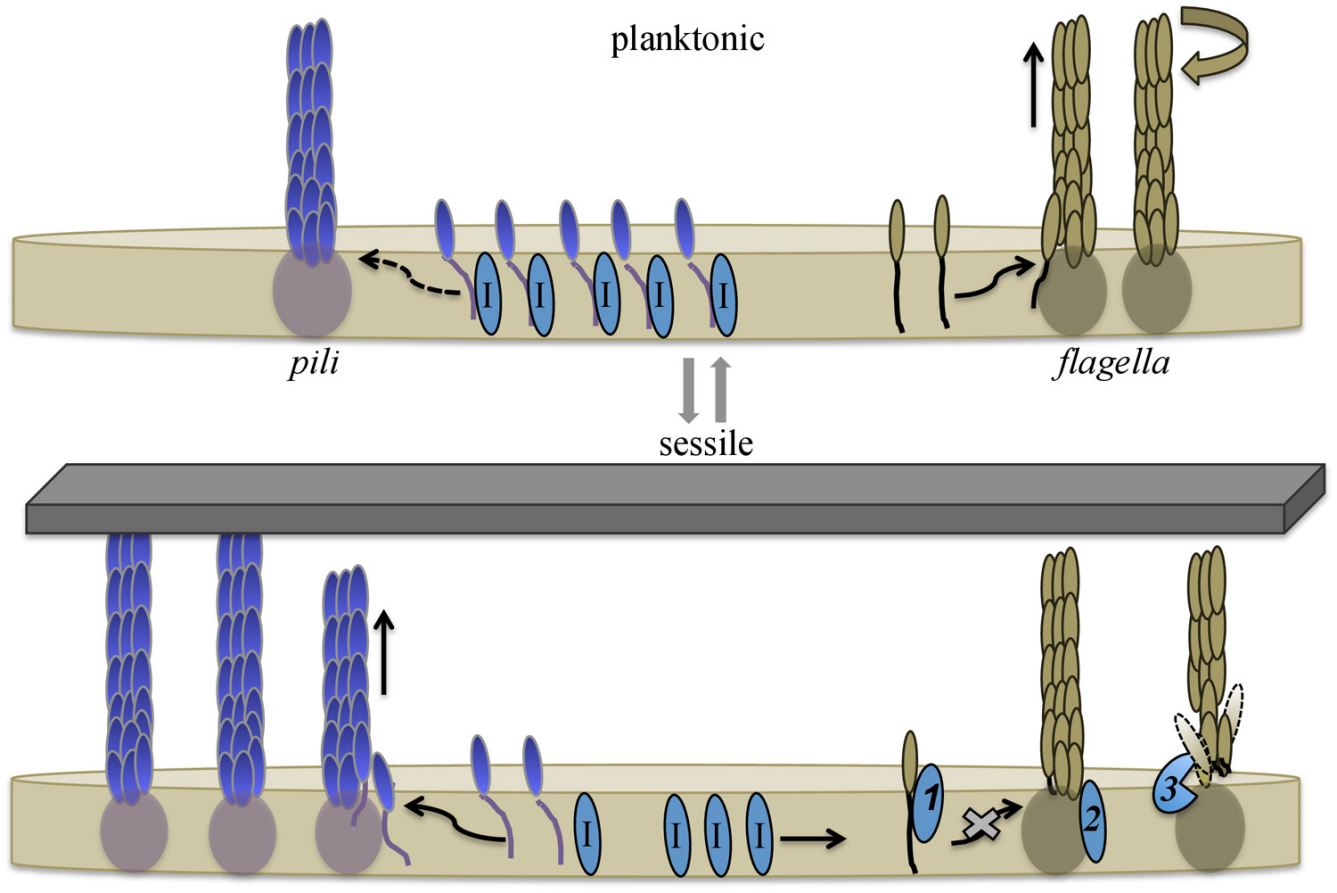
Model for pilin-mediated inhibition of swimming motility. During planktonic growth *H. volcanii* cells synthesize flagellins that are readily incorporated into flagella, supporting swimming motility. Cells also express type IV pilins, which are incorporated into pili at a slow rate. The H-domain of membrane-associated pilins interacts with, and hence sequesters, a protein that directly or indirectly inhibits flagella motility. Upon adhesion, pilus-assembly kinetics shift, and the incorporation of pilins into pili increases, depleting the membrane of pilins and releasing the inhibitor proteins. The released inhibitors interfere with flagella biosynthesis and/or stability. Taken together, this allows cells to rapidly respond to environmental conditions that favor biofilm formation over motility. Three possible mechanisms through which an inhibitor might hinder swimming motility are: (1) direct interaction with flagellins, preventing the incorporation of subunits into the flagellum; (2) inactivation of a flagella-biosynthesis component(s); or (3) degradation/destabilization of the flagella. Dashed arrow indicates limited incorporation into pili. Putative inhibitor proteins are labeled with I. Image modified from (Esquivel and Pohlschroder, 2014).

Contrary to the models described above, deleting *H. volcanii pilB3* and *pilC3,* homologs of *aapE* and *aapF*, respectively, has no obvious effect on motility. More surprising, deleting all six *H. volcanii* genes that encode PilA pilins (Δ*pilA*[*1-6*]) results in cells with a severe motility defect (Esquivel and Pohlschroder, 2014). Moreover, expressing any one of these pilins in *trans* in a Δ*pilA*[*1-6*] deletion mutant restores swimming motility (**Table 1**). However, when the PilA1Hybrid (See Adhesion section above) is expressed in the Δ*pilA*[*1-6*] strain, motility is not restored, indicating that these pilins, and more specifically, their conserved hydrophobic domain, play an important role in regulating flagella-dependent motility (Esquivel and Pohlschroder, 2014). Consistent with this hypothesis, in a Δ*pilA*[*1-6*] strain, heterologous expression of a FlgA1Hybrid in which the flagellin hydrophobic domain is replaced with the conserved PilA H-domain results in a restoration of motility, albeit to less than wild-type levels, despite the fact that this hybrid flagellin does not complement a motility defect caused by the deletion of *flgA1.* Furthermore, overexpressing any one of the *pilA* genes in a wild-type strain causes hypermotility (Esquivel and Pohlschroder, 2014). Considering that these pilins do not directly interact with either the flagellins or the flagellum, they appear to regulate motility within the membrane, perhaps by sequestering a protein that inhibits flagella biosynthesis (**Figure 5**). The regulation of flagella-dependent motility by proteins required for biofilm formation is reminiscent of the inhibition of swimming motility by the *B. subtilis* oligosaccharyltransferase, a bifunctional enzyme that is not only critical for EPS biosynthesis, it also acts like a clutch while interacting with the flagellum (Blair et al., 2008).

Finally, although there is not yet *in vivo* data supporting this hypothesis, as noted above, we know most archaea use a single prepilin peptidase, PibD to process both flagellins and pilins before they are incorporated into a filament (Albers et al., 2003; Szabó et al., 2007; Tripepi et al., 2010). Thus, perhaps when prokaryotic cells attach to a surface, and pilin precursor expression increases, the PibD available for processing the flagellins might become more limited. Alternatively, in organisms such as *M. maripaludis* that have two PibD homologs, the availability of a second peptidase might allow the cells to shift more rapidly between high levels of flagella and high levels of type IV pili. While much work needs to be done to determine the details of the various regulatory mechanisms outlined here, the fact that the biosynthesis and functions of the flagella and type pili are regulated at several different levels underscores the importance of an ability to quickly transition from a planktonic to a sessile state and *vice versa*.

## Concluding Remarks

During the past decade, biochemical and molecular biological studies combined with sophisticated microscopy, on a diverse set of archaeal models, have clearly demonstrated the critical importance of the evolutionarily conserved type IV pili in archaeal biofilm formation. These studies have also confirmed that the core components of the type IV pilus biosynthesis machinery are conserved across prokaryotic domains, and, conversely, revealed that, as compared to the bacterial filaments, there are novel aspects to the biosynthesis, regulation, and functions of archaeal type IV pili. A number of unique, previously unidentified, components of the archaeal type IV pilus biosynthesis machinery have been identified recently, and detailed analyses during the coming decade will be crucial in determining the roles these proteins play in pilus assembly. These analyses will include advanced approaches such as co-purifications coupled with sophisticated mass spectrometry as well as insertional mutagenesis as exemplified by the transposon screen that was recently developed for use in *H. volcanii* (Kiljunen et al., 2014). Such approaches should facilitate the identification of as yet unknown, but likely present, proteins that are critical for pilus assembly or function. Forthcoming studies of archaeal type IV pili will also focus on determining the significance of distinct sets of type IV pili that are commonly found within a single archaeon, including pili for which assembly depends on distinct sets of PilB and PilC paralogs. Among the prokaryotes that have been investigated, archaeal, as well as bacterial, species, can express up to six distinct PilB and PilC sets of paralogs. Furthermore, type IV pili composed of distinctly unrelated pilin subunits can still depend upon the same core components for assembly.

To date, the functions of the vast majority of predicted archaeal type IV pilins, including those encoded by the genomes of model archaeal systems, remain undetermined, perhaps because the conditions under which these pilins function have not been experimentally replicated. For instance, these predicted pilins may be the subunits of pili that are required for attachment to surfaces that have not yet been assayed. Crucial insights into the roles these pilins play in adhesion, microcolony formation, biofilm maturation, and dispersal can be gained by performing *in vivo* assays, along with RNAseq and mass spectrometry on wild-type cells, as well as specific mutants, that are isolated during various stages of biofilm formation; currently, assays are performed predominantly on planktonic cells. The dynamics of pilus diversity on the cell surface, the dynamics of pilus subunit composition, as well as changes in the post-translational modifications of pilins during different stages of biofilm formation are not only poorly understood in archaea, but also in bacteria. Finally, while these studies are currently in their infancy, the tools needed to study the regulatory mechanisms that control transitions between planktonic to sessile cell states, where type IV pili appear to play key roles in archaea, are already available. Despite the discovery of previously unknown archaeal type IV pilus biosynthesis components, the molecular machinery involved in assembling type IV pilus-like structures in archaea appears to be significantly less complex than its counterpart in bacteria, simplifying detailed analysis of the molecular machinery involved in type IV pilus biosynthesis. Hence, future analyses of archaeal type IV pilus-biosynthesis may not only reveal further details about archaeal pilus-biosynthesis but about pilus biosynthesis in general. Comparisons between various archaeal systems, and between archaeal and bacterial systems, will help elucidate the types of adaptations that have allowed prokaryotes to thrive under a diverse variety of environmental conditions and may also provide useful information to aid in the development of a diverse set of industrial applications.

Decades of research in bacteria have provided a solid foundation for the study of archaeal type IV pili. Now, studies of the archaeal type IV pili will lead to important insights into the evolutionary history of these ancient cell surface structures and may lead to the identification of novel functions and regulatory mechanisms, which might also set in motion new lines of research on bacterial pili.

## Conflict of Interest Statement

The authors declare that the research was conducted in the absence of any commercial or financial relationships that could be construed as a potential conflict of interest.
